# High prevalence of plasmid-mediated quinolone resistance determinants in commensal members of the *Enterobacteriaceae* in Ho Chi Minh City, Vietnam

**DOI:** 10.1099/jmm.0.010033-0

**Published:** 2009-12

**Authors:** Le Thi Minh Vien, Stephen Baker, Le Thi Phuong Thao, Le Thi Phuong Tu, Cao Thu Thuy, Tran Thi Thu Nga, Nguyen Van Minh Hoang, James Iain Campbell, Lam Minh Yen, Nguyen Trong Hieu, Nguyen Van Vinh Chau, Jeremy Farrar, Constance Schultsz

**Affiliations:** 1 Oxford University Clinical Research Unit, Hospital for Tropical Diseases, Ho Chi Minh City, Vietnam; 2 Nuffield Department of Clinical Medicine, John Radcliffe Hospital, University of Oxford, Oxford, UK; 3 Hospital for Tropical Diseases, Ho Chi Minh City, Vietnam; 4 Hung Vuong Hospital, Ho Chi Minh City, Vietnam; 5 Center for Poverty-related Communicable Diseases (CPCD), Academic Medical Center, University of Amsterdam, Amsterdam, The Netherlands

## Abstract

Antimicrobial-resistant pathogenic members of the *Enterobacteriaceae* are a well-defined global problem. We hypothesized that one of the main reservoirs of dissemination of antimicrobial resistance genes in Vietnam is non-pathogenic intestinal flora, and sought to isolate antimicrobial-resistant organisms from hospitalized patients and non-hospitalized healthy individuals in Ho Chi Minh City. The results identified substantial faecal carriage of gentamicin-, ceftazidime- and nalidixic acid-resistant members of the *Enterobacteriaceae* in both hospitalized patients and non-hospitalized healthy individuals. A high prevalence of quinolone resistance determinants was identified, particularly the *qnrS* gene, in both community- and hospital-associated strains. Furthermore, the results demonstrated that a combination of quinolone resistance determinants can confer resistance to nalidixic acid and ciprofloxacin, even in the apparent absence of additional chromosomal resistance mutations in wild-type strains and laboratory strains with transferred plasmids. These data suggest that intestinal commensal organisms are a significant reservoir for the dissemination of plasmid-mediated quinolone resistance in Ho Chi Minh City.

## INTRODUCTION

Pathogenic enteric bacteria that exhibit antimicrobial resistance are a widespread phenomenon and arguably constitute a global epidemic. Whilst the depth of knowledge regarding antimicrobial-resistant organisms isolated from patients with infection or circulating in the hospital environment is broad, less is known about antimicrobial-resistant organisms that are disseminated in the community. Furthermore, little is known about the antimicrobial resistance patterns of community-acquired organisms that circulate in developing countries where antimicrobials are available without prior consultation with a physician.

Quinolones and fluoroquinolones are groups of antimicrobial compounds that are commonly used for the treatment of many bacterial infections. However, multiple studies have highlighted that, in recent years, resistance to fluoroquinolones has increased globally, particularly in members of the *Enterobacteriaceae* such as *Salmonella* and *Klebsiella* species (Chau *et al.*, 2007; Strahilevitz *et al.*, 2007; Wang *et al.*, 2008). Quinolones target the DNA gyrase and topoisomerase IV enzymes of the bacterial cell, thus preventing DNA replication (Higgins *et al.*, 2003). The main resistance mechanisms to quinolones are mutations in the *gyrA* and *parC* genes that alter the conformation of target amino acid residues within the protein (Jacoby, 2005). The more recent discovery and rapid dissemination of plasmid-mediated quinolone resistance (PMQR) genes has further highlighted the problem of quinolone and fluoroquinolone resistance and increased our understanding of resistance mechanisms associated with these antibacterial compounds (Robicsek *et al.*, 2006a).

The first PMQR gene to be described was named *qnrA* and encodes a 218 aa pentapeptide repeat protein that is capable of protecting the DNA gyrase from the activity of quinolones (Robicsek *et al.*, 2006a; Tran *et al.*, 2005a, b). Since the discovery of QnrA, two related PMQR proteins have been described; these proteins are thought to act in a manner comparable to QnrA, as they share 40 and 59 % amino acid similarity and have been named QnrB and QnrS, respectively (Hata *et al.*, 2005; Jacoby *et al.*, 2006). *In vitro* acquisition of a Qnr determinant through conjugation confers a 16–125-fold increase in the MIC, depending on the nature of the donor strain and the quinolone tested (Robicsek *et al.*, 2006a).

Two additional PMQR determinants have also been described, which act using two mechanisms distinct from that of the Qnr proteins. The first is the *aac(6′)-Ib-cr* gene, which encodes a variant aminoglycoside acetyltransferase (Melano *et al.*, 2003; Park *et al.*, 2006). This gene harbours two individual base pair substitutions, which result in the enzyme being able to acetylate ciprofloxacin and norfloxacin, reducing the activity of the fluoroquinolone and therefore increasing the MIC two- to fourfold (Robicsek *et al.*, 2006b). The second is the *qepA* gene, which encodes a major facilitator subfamily quinolone-specific efflux pump (Perichon *et al.*, 2007). Experimentally, the QepA protein does not alter the MICs of ampicillin, erythromycin, kanamycin or tetracycline, but it does decrease susceptibility to norfloxacin, enrofloxacin and ciprofloxacin by up to 64-fold (Yamane *et al.*, 2007).

All of the Qnr determinants have been identified in clinical *Enterobacteriaceae* isolates; the majority of reported strains are isolates of *Salmonella* species and *Klebsiella pneumoniae* (Cattoir *et al.*, 2007b; Gay *et al.*, 2006; Mendes *et al.*, 2008; Strahilevitz *et al.*, 2007). The *aac(6′)-Ib-cr* gene has also been reported in pathogenic bacteria and, like the *qnr* genes, has been found worldwide. The *qepA* gene has been reported in members of the *Enterobacteriaceae* in Japan, China and France (Cattoir *et al.*, 2008b; Ma *et al.*, 2009; Yamane *et al.*, 2008). In this study, we attempted to demonstrate the frequency of carriage of antimicrobial-resistant members of the *Enterobacteriaceae* in patients and healthy individuals living in Ho Chi Minh City, Vietnam. We determined the prevalence of PMQR genes in these strains and their effect on the MIC in wild-type strains with and without additional resistance-associated chromosomal mutations, as well as in laboratory strains transformed with isolated plasmid DNA.

## METHODS

### Bacterial isolation and identification.

The strains were collected from two distinct study populations comprising strains obtained from patients admitted to hospital (hospital strains) and those from healthy volunteers from the local community (community strains). Hospital strains were collected from 194 patients who were admitted to the Tetanus Intensive Care Ward at the Hospital for Tropical Diseases in Ho Chi Minh City over the periods of May–October 2004 and June–November 2005. Swabs for culture were taken from the axilla, nose, sputum and anus of all patients on admission and twice weekly.

The community strains were collected from stool samples from 27 healthy adults and 77 children (5–14 years) living in Ho Chi Minh City, who participated in a typhoid vaccine study in 2005 and 2006, and from nasal and rectal swabs from 100 consecutive 1–3-day-old healthy neonates, born after uncomplicated pregnancies on a general obstetrics ward in 2006. None of the individuals (including the mothers of the neonates) had had any known contact with antibiotics (prescribed or otherwise) for 4 weeks prior to sample collection.

Samples were cultured on MacConkey medium with and without supplementation of gentamicin (8 μg ml^−1^), ceftazidime (2 μg ml^−1^) or nalidixic acid (16 μg ml^−1^) (Sigma-Aldrich). All colony morphologies grown on MacConkey agar supplemented with antibiotics were Gram stained. All isolates confirmed to be Gram-negative and oxidase-negative were identified using an API 20E system (bioMérieux). For hospitalized patients, the first isolate growing on each of the selective agars, typically ranging from 2 days to 1 week after admission, was included in the study.

### Antimicrobial susceptibility testing.

The susceptibility to gentamicin, amikacin, ceftazidime, piperacillin–tazobactam, imipenem and nalidixic acid of members of the *Enterobacteriaceae* growing on selective MacConkey agars was determined using a disc diffusion method on Mueller–Hinton agar plates, according to Clinical and Laboratory Standards Institute (CLSI) guidelines (CLSI, 2007). Strains that were identified as resistant to ceftazidime were subjected to further phenotypic tests to confirm extended-spectrum *β*-lactamase (ESBL) production, using discs containing only cefotaxime (30 μg) and ceftazidime (30 μg) and both antimicrobials combined with clavulanic acid (10 μg), according to CLSI guidelines (CLSI, 2007). MICs were measured using E-test (AB Biodisk).

### PCR and DNA sequencing.

Genomic DNA was isolated from all isolated members of the *Enterobacteriaceae* using a Wizard Genomic DNA Preparation kit (Promega), and PCR amplification of the *gyrA*, *parC*, *qnrA*, *qnrB*, *qnrS*, *qepA* and *aac(6′)-Ib* genes was performed using the primers outlined in Table 1.

PCR amplification was carried out for 35 cycles at 94 °C for 30 s, 55 °C [*gyrA*, *qnrB*, *parC*, *qepA* and *aac(6′)-Ib*] or 48 °C (*qnrA* and *qnrS*) for 30 s, and 72 °C for 30 s. Amplification was performed using a DNA Engine Tetrad 2 (Bio-Rad) and BioTaq polymerase (Bioline). The resulting PCR amplicons were examined by electrophoresis and UV visualization on 2 % agarose gels containing ethidium bromide.

Amplicons produced from all strains specific for the *gyrA*, *parC*, *qnrS*, *qepA* and *aac(6′)-Ib* genes were sequenced with the same primers used for amplification. The forward and reverse strands of all PCR products were sequenced using BigDye terminators on a CEQ8000 DNA sequencer (Beckman Coulter).

The genetic environment of the *qnrS* gene was identified by cloning plasmid digestions in pUC18 and sequencing. DNA sequences were edited and analysed using Vector NTI software and compared with other sequences using blastn on the NCBI sequence database.

### Molecular typing.

Strain differentiation was performed to identify clonally related strains isolated from different individuals. All hospital strains and all of the *qnr-* or *aac(6′)-Ib-cr*-positive community strains were typed by random amplified polymorphic DNA (RAPD) PCR using three different primers (Table 1) (Schultsz *et al.*, 1997; Versalovic *et al.*, 1991). All hospital strains from each 6-month period and all community strains were processed simultaneously for each primer, and electrophoresis was performed under identical conditions on the same day. RAPD patterns were clustered and interpreted by combining the resulting amplification patterns of all three primers and analysed using Bionumeric software (Applied Maths). Two isolates were considered to be clonally related when they had RAPD patterns that were identical. Isolates of a given species differing by one or two bands for all three primers combined were considered to be variants of a given type. This was based on similarities in patterns of multiple isolates obtained from individual patients, by visualization and computer-based analysis using Bionumerics.

### Plasmid extraction and manipulation.

Plasmid DNA for electrotransformation was extracted using a Midiprep Plasmid DNA Extraction kit, following the manufacturer's recommendations (Qiagen). *Escherichia coli* TOP10 cells (Invitrogen) were transformed with isolated DNA using a Bio-Rad gene pulser, under conditions recommended by the manufacturer (Invitrogen). Transformants were selected on Luria–Bertani medium supplemented with 0.012 μg ciprofloxacin ml^−1^.

Conjugation was performed at a 1 : 1 ratio in liquid cultures by static incubation overnight at 37 °C. *E. coli* J53 (sodium azide resistant) was used as the recipient. Transconjugants were selected by plating onto Luria–Bertani medium supplemented with 0.03 μg ciprofloxacin ml^−1^ and 100 μg sodium azide ml^−1^.

Plasmid DNA for sizing and visualization was extracted using an alkaline lysis procedure, as described by Kado & Liu (1981). The resulting plasmid DNA was separated by electrophoresis in 0.7 % agarose gels made with 1× E buffer (Kado & Liu, 1981). Gels were run at 90 V for 3 h, stained with ethidium bromide and photographed. *E. coli* 39R861 containing plasmids of 7, 36, 63 and 147 kbp was used for sizing plasmid extractions on agarose gels.

## RESULTS AND DISCUSSION

### Isolation of antimicrobial-resistant commensal *Enterobacteriaceae*

A total of 194 hospitalized patients were tested over the study period. We isolated 70 *E. coli* strains, 123 *K. pneumoniae* strains and 29 other members of the *Enterobacteriaceae*, comprising *Citrobacter* species, *Enterobacter cloacae*, *Proteus mirabilis*, *Klebsiella ornithinolytica*, *Pantoea* species and a *Vibrio fluvialis*, resistant to gentamicin, ceftazidime or nalidixic acid, or a combination of these antimicrobials, from these patients. On the basis of RAPD results, we identified 53 unique *E. coli* strains, 62 unique *K. pneumoniae* strains and 16 other unique members of the *Enterobacteriaceae*.

The community group totalled 204 people and included 27 healthy adults, 77 healthy children and 100 healthy neonates. On the basis of culture on selective media containing gentamicin, ceftazidime or nalidixic acid, we isolated 340 resistant *E. coli* strains, 45 resistant *K. pneumoniae* strains and 28 other resistant members of the *Enterobacteriaceae*. Analysis of the RAPD patterns showed that all strains isolated from the different individuals were unique (data not shown). However, on the basis of typing and resistance patterns, we were able to isolate bacteria displaying a consistent RAPD pattern from consecutive isolates obtained over up to 14 days in 18 individuals, thus indicating carriage (data not shown).

We isolated organisms that were resistant to gentamicin, ceftazidime or nalidixic acid, or a combination of these, from 93 % (25/27) of the healthy adults, 92 % (71/77) of the healthy children, 64 % (64/100) of the healthy neonates and 68 % (132/194) of the tetanus patients. Combining the data for all 544 unique, resistant strains, 42 % of organisms were resistant to ceftazidime, 63 % were resistant to gentamicin and 74 % were resistant to nalidixic acid. All of the organisms that were resistant to ceftazidime were confirmed to be ESBL producers.

These results showed that the dissemination of resistant enteric bacteria is rife in the hospital and the community in Ho Chi Minh City. Antimicrobials are available without prescription in Vietnam; therefore, it is tempting to suggest that this is a primary source of selection for resistant organisms. However, none of the healthy individuals had had any antimicrobial therapy for at least 4 weeks prior to sample collection. The use of antimicrobials in the production of meat and vegetables is another potential major source of the ongoing selection of resistant organisms (Stobberingh & van den Bogaard, 2000; Teuber, 2001). In recent reports, investigators have shown intestinal colonization with fluoroquinolone-resistant and ESBL-producing *E. coli* in healthy schoolchildren in Latin America and in vegetarians in the USA (Pallecchi *et al.*, 2007a, b; Sannes *et al.*, 2008). These data suggest exposure to particular antimicrobials to such an extent as to maintain resistant organisms in the intestinal flora and/or transmission of resistant strains from individuals exposed to these agents followed by persistent carriage.

### Amplification of plasmid-mediated quinolone resistance genes

All strains were subjected to PCR to amplify the individual *qnrA*, *qnrB*, *qnrS*, *qepA* and *aac(6′)-Ib-cr* genes. The majority of strains that generated a PMQR amplicon were positive for a single PMQR determinant (Table 2). Variability in PMQR determinant content was observed for a limited number of strains with identical RAPD patterns among the hospital strains. Based on a combination of RAPD pattern and PMQR content of strains, we identified 55 unique *E. coli* strains, 66 *K. pneumoniae* strains and 18 other members of the *Enterobacteriaceae* in this group.

Of the *K. pneumoniae* hospital strains, 78.8 % (52/66) produced amplicons for the *qnrS* gene (Table 2). The overall numbers of PMQR genes identified were lower in the community strains compared with the hospital strains; none the less, 9.4 % (32/340) of the *E. coli* community strains were PCR positive for the *qnrS* gene. We were able to detect *aac(6′)-lb-cr*-positive *E. coli* and *K. pneumoniae* from both hospital and community strains, albeit at a comparatively low frequency (Table 2). Of the 154 strains containing PMQR determinants, 98 (63.6 %) tested ESBL positive, including 59/112 (52.7 %) *qnrS*-positive strains.

The sequences for all of the amplicons of the *qnrS* fragment were indistinguishable and demonstrated 100 % sequence identity with *qnrS1* from *K. pneumoniae* strain 052250 (GenBank accession no. EF683584). The sequences of the *qnrA* and *qnrB* amplicons and the single *qepA* PCR amplicon demonstrated 100 % identity to *qnrA1* and *qnrB1* from *K. pneumoniae* plasmid pMG252 (GenBank accession no. DQ831140) and *K. pneumoniae* plasmid pMG298 (GenBank accession no. DQ351241) and *E. coli* plasmid pHPA (GenBank accession no. AB263754), respectively.

These results indicated a very high prevalence of *qnr* genes, in particular the *qnrS* gene, in commensal isolates in Ho Chi Minh City. Recent reports showing *qnr* genes originating from waterborne bacteria may explain the dissemination of *qnr* genes in commensal bacteria (Cattoir *et al.*, 2007a, 2008a). Faecal–oral transmission may facilitate exchange of *qnr* genes between waterborne and intestinal bacteria in the human host.

### Effect of *gyrA* and *parC* mutations and PMQR genes on susceptibility to nalidixic acid and ciprofloxacin

Chromosomal quinolone resistance in *E. coli* and *K. pneumoniae* is determined predominantly by mutations at codons 83 and 87 in the *gyrA* gene. The combinatorial effect of harbouring one or more of the PMQR genes and the mutations in the *gyrA* gene on the susceptibility of the bacteria to nalidixic acid and ciprofloxacin was assessed in all unique hospital strains and all PMQR-positive community strains.

In the 55 unique hospital *E. coli* strains, 10 (18.2 %) had a single mutation at codon 83, none had a single mutation at codon 87, and 38 (69.1 %) had a double mutation in the *gyrA* gene. In 42 community *E. coli* strains, six (14.3 %) had a single mutation at codon 83 (Ser→Leu or Ala), none had a single mutation at codon 87 and 13 (31 .0%) had a double mutation (^83^Ser→Leu or Ala; ^87^Asp→Asn).

In the 66 hospital *K. pneumoniae* strains sequenced, Ser was mutated to Tyr, Ile and Phe at position 83 in eight (12.1 %), seven (10.6 %) and two (3.0 %) strains, respectively. Of the 15 community *K. pneumoniae* strains, Ser was mutated to Tyr at codon 83 in one strain (6.7 %). Asp was mutated to Ala at codon 87 in five (7.6 %) and one (6.7 %) hospital and community *K. pneumoniae* strains, respectively.

The combinatorial effects of the various PMQR genes and associated *gyrA* mutations on the MICs for nalidixic acid and ciprofloxacin are shown in Table 3 (hospital strains) and Table 4 (community strains). The biggest MIC increases associated with a single gene were associated with *qnrS* or *aac(6′)-Ib-cr*. We obtained two strains carrying the *qnrS*, *qnrB* and *aac(6′)-Ib-cr* genes with full resistance to nalidixic acid and ciprofloxacin, in the absence of known selective mutation in the *gyrA* gene (Table 3). The effect of PMQR genes on the resulting MIC to fluoroquinolones thus appeared to be reliant on the number and type of PMQR genes carried by the bacteria. The co-existence of *qnrB* and *qnrS* genes on two different plasmids within a strain has been shown previously. This combination did not have any additional effect on resistance to nalidixic acid (Hu *et al.*, 2008), in contrast to our strains. We hypothesized a combinatorial effect, due to the proteins working in an independent manner: QnrB and QnrS protecting DNA gyrase and Aac(6*′*)-Ib-cr modifying the fluoroquinolone. However, as Aac(6*′*)-Ib-cr is thought not to modify quinolones such as nalidixic acid, we cannot currently rule out any possible effects of other quinolone resistance mechanisms, such as non-specific efflux pump activity.

None of the PMQR-positive strains with a MIC of ≥8 mg nalidixic acid l^−1^ or ≥1 mg ciprofloxacin l^−1^ demonstrated any mutation at positions 80 and 84 of the *parC* gene, which have been shown to decrease the susceptibility of *E. coli* and *K. pneumoniae* to both nalidixic acid and ciprofloxacin (Brisse & Verhoef, 2001; Vila *et al.*, 1996).

### Characterization of *qnrS*-containing plasmids

We selected *qnrS* PCR-positive strains *E. coli* E66An (hospital), *K. pneumoniae* K1HV (community) and *K. pneumoniae* K18An (hospital) for further analysis (Table 5). We were unable to transfer nalidixic acid resistance by conjugation from either of the two *K. pneumoniae* strains into the *E. coli* recipient. However, we were able to transform *E. coli* TOP10 with purified plasmid DNA from both strains and select transformants on the basis of ciprofloxacin resistance. In contrast, we were able to transfer nalidixic acid resistance via both conjugation and transformation from *E. coli* E66An into another *E. coli* strain.

**Fig. 1. f1:**
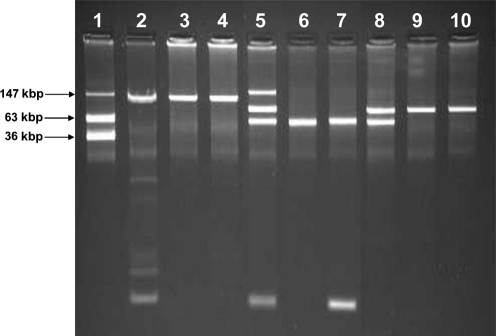
Agarose gel electrophoresis of alkaline plasmid lysis preparation from wild-type, transconjugant and electrotransformant *E. coli* and *K. pneumoniae* strains. Lanes: 1, *E. coli* 39R861; 2, *K. pneumoniae* 1HV; 3, *E. coli* transformant 1 1HV; 4, *E. coli* transformant 2 1HV; 5, *K. pneumoniae* 18An; 6, *E. coli* transformant 1 18An; 7, *E. coli* transformant 2 18An; 8, *E. coli* 66An; 9, *E. coli* transformant E66An; 10, *E. coli* transconjugant E66An.

Comparison of the transformants derived from plasmid DNA isolated from both *K. pneumoniae* strains and the transformant and transconjugant derived from *E. coli* E66An indicated that sizes and resistance profiles varied across the different plasmids containing the *qnrS* gene (as confirmed by PCR) (Fig. 1, lanes 3, 4, 6 and 7; Table 5).

The transconjugant strain derived from *E. coli* E66An, but not the transformants derived from the *K. pneumoniae* strains, additionally showed that ESBL production had been transferred alongside *qnrS* (Table 5). The correlation between *qnrA* or *qnrB* and ESBL genes is well known (Iabadene *et al.*, 2008; Jiang *et al.*, 2008; Oktem *et al.*, 2008; Szabo *et al.*, 2008; Wang *et al.*, 2008), whilst the relationship between *qnrS* and other resistance genes is less well described. ESBL production was observed in only 52.7 % of the *qnrS*-positive strains in our study, and our transformation and conjugation experiments confirmed that *qnrS* genes can be located on plasmids that do not contain ESBL genes.

Sequence analysis of the region surrounding the *qnrS* gene in this *E. coli* strain demonstrated 100 % sequence identity to a previously sequenced transposon in plasmid pK245 in *K. pneumoniae* strain NK245, from a patient with hospital-acquired urinary tract infection in Taiwan (Chen *et al.*, 2006). The *qnrS* gene appears to ‘piggy back’ on a mobile element, and selection may occur on the basis of the presence of other antimicrobial resistance genes (Chen *et al.*, 2006). This is also suggested by the apparent redundant nature of the PMQR genes in those strains that have a double mutation in the *gyrA* gene. The variability in size of plasmids harbouring the *qnrS* gene, observed in our study, suggests that the genetic element carrying the *qnrS* gene has been mobilized onto numerous plasmids of different size.

Our study indicates that commensal organisms may represent the greatest reservoir and source of dissemination of plasmid-mediated antimicrobial resistance genes, such as *qnrS*, in Vietnam.

## Figures and Tables

**Table 1. t1:** Primers used in this study na, Not applicable.

**Target gene**	**Gene size (bp)**	**Primer name**	**Predicted size of amplicon (bp)**	**Primer sequence (5′→ 3′)**
*qnrA*	657	QnrA-F	627	TCAGCAAGAGGATTTCTCA
		QnrA-R		GGCAGCACTATTACTCCCA
*qnrB*	681	QnrBm-F	264	GGMATHGAAATTCGCCACTG
		QnrBm-R		TTTGCYGYYCGCCAGTCGAA
*qnrS*	657	QnrS-F	491	ATGGAAACCTACAATCATAC
		QnrS-R		AAAAACACCTCGACTTAAGT
*aac(6′)-Ib*	519	Aac(6*′*)-Ib-F	482	TTGCGATGCTCTATGAGTGGCTA
		Aac(6*′*)-Ib-R		CTCGAATGCCTGGCGTGTTT
*qepA*	986	QepA_F	199	GCAGGTCCAGCAGCGGGTAG
		QepA_R		CTTCCTGCCCGAGTATCGTG
*gyrA*	2628	GyrA-F	625	CGACCTTGCGAGAGAAAT
		GyrA-R		GTTCCATCAGCCCTTCAA
*parC* (*E. coli*)	2258	parC_E_F	395	AAACCTGTTCAGCGCCGCATT
		parC_E_R		GTGGTGCCGTTAAGCAAA
*parC* (*K. pneumoniae*)	2258	parC_K_F1	389	CTGAATGCCAGCGCCAAATT
		parC_K_R1		TGCGGTGGAATATCGGTCGC
RAPD	na	ERIC1	na	ATGTAAGCTCCTGGGGATTCAC
		ERIC2		AAGTAAGTGACTGGGGTGAGCG
		TT3		GGCGAGGAGCG

**Table 2. t2:** Numbers of unique bacterial isolates from the hospital and community groups carrying a fluoroquinolone resistance determinant

**Source of bacterial isolate**	**Bacterial species (*n*)**	**PCR positive strains [*n* (%)]**					
		***qnrA***	***qnrB***	***qnrS***	***aac(6′)-Ib-cr***	***qepA***	>1 PMQR*
Hospital group	*E. coli* (55)	5 (9.0)	1 (1.8)	5 (9.0)	9 (16.4)	1 (1.8)	2 (3.6)
	*K. pneumoniae* (66)	4 (6.1)	8 (12.1)	52 (78.8)	11 (16.7)	0 (0)	11 (16.7)
	Other† (18)	6 (33.3)	8 (44.4)	6 (33.3)	0 (0)	0 (0)	4 (22.2)
Community group	*E. coli* (340)	2 (0.6)	0 (0)	32 (9.4)	9 (2.6)	0 (0)	1 (0.3)
	*K. pneumoniae* (45)	0 (0)	0 (0)	15 (33.3)	1 (2.2)	0 (0)	1 (2.2)
	Other‡ (28)	1 (3.6)	2 (7.1)	2 (7.1)	1 (3.6)	0 (0)	1 (3.6)

*PCR positive for more than one PMQR gene inclusive of data in the rest of the table. †Other bacterial species isolated from patients included three *Enterobacter cloacae*, three *Citrobacter youngae*, three *Citrobacter freundii*, two *P. mirabilis*, one *Citrobacter* species and one *Citrobacter koseri*. ‡Other bacterial species isolated from the community included two *Citrobacter* species, one *Enterobacter cloacae* and one *Klebsiella terrigena*.

**Table 3. t3:** Resulting MIC range (mg l^−1^) of *E. coli* and *K. pneumoniae* strains isolated from hospitalized patients, associated with a variety of combinations of PMQR genes and mutations in the *gyrA* gene None of the strains contained mutations at codons 80 and 84 of the *parC* gene. NAL, Nalidixic acid; CIP, ciprofloxacin.

**PCR-positive gene**	**Strain (*n*)**	**Number of mutations in *gyrA***								
		**0**			**1**			**2**		
		***n***	**NAL**	**CIP**	***n***	**NAL**	**CIP**	***n***	**NAL**	**CIP**
None	*E. coli* (36)	5	3–6	0.012–0.016	10	>256	0.19–0.38	21	>256	>32
	*K. pneumoniae* (9)	3	4–32	0.06–1	4	>256	6–>32	2	>256	>32
*qnrA*	*E. coli* (5)	0			0			5	>256	>32
	*K. pneumoniae* (1)	1	8	0.25	0			0		
*qnrB*	*E. coli* (1)	0			0			1	>256	>32
	*K. pneumoniae* (1)	1	12	0.38	0			0		
*qnrS*	*E. coli* (4)	2	24, 32	0.38				2	>256	>32
	*K. pneumoniae* (42)	38*	6–64	0.38–4	3	>256	3–8	1	>256	>32
*aac(6′)-Ib-cr*	*E. coli* (8)†	0			0			8	>256	>32
*qnrA+qnrS*	*K. pneumoniae* (2)	2	24, 32	0.75, 1	0			0		
*qnrB+aac(6′)-Ib-cr*	*K. pneumoniae* (3)	0			1	>256	>32	2	>256	>32
*qnrS+aac(6′)-Ib-cr*	*E. coli* (1)	0			0			1	>256	>32
	*K. pneumoniae* (3)	2	16, 32	2	1	>256	>32	0		
*qnrA+qnrS+aac(6′)-Ib-cr*	*K. pneumoniae* (1)	1	16	3	0			0		
*qnrB+qnrS+aac(6′)-Ib-cr*	*K. pneumoniae* (4)	2	>256	>32	0			2	>256	>32

*NAL: MIC_50_=16, MIC_90_=32; CIP: MIC_50_=0.75, MIC_90_=1.5. †Included one *qepA* positive strain.

**Table 4. t4:** Resulting MIC range (mg l^−1^) of *E. coli* and *K. pneumoniae* strains isolated from healthy individuals, associated with a variety of combinations of PMQR genes and mutations in the *gyrA* gene None of the strains contained mutations at codons 80 and 84 of the *parC* gene. NAL, Nalidixic acid; CIP, ciprofloxacin.

**PCR positive gene**	**Strains (*n*)**	**Number of mutations in *gyrA***								
		**0**			**1**			**2**		
		***n***	**NAL**	**CIP**	***n***	**NAL**	**CIP**	***n***	**NAL**	**CIP**
*qnrA*	*E. coli* (1)	0			0			1	>256	8
*qnrS*	*E. coli* (31)	22*	3–64	0.19–1.5	5	>256	1.5–32	4	>256	>32
	*K. pneumoniae* (14)	14†	8–48	0.5–1.5	0			0		
*aac(6′)-Ib-cr*	*E. coli* (9)	0			1	>256	2	8	>256	>32
*qnrA+qnrS*	*E. coli* (1)	1	6	0.5	0			0		
*qnrS+aac(6′)-Ib-cr*	*K. pneumoniae* (1)	0			0			1	>256	>32

*NAL: MIC_50_=12, MIC_90_=48; CIP: MIC_50_=0.38, MIC_90_=0.75. †NAL: MIC_50_=12, MIC_90_=16; CIP: MIC_50_=0.75, MIC_90_=1.5.

**Table 5. t5:** Resulting susceptibility patterns of wild-type, transconjugant and electrotransformant *E. coli* and *K. pneumoniae* strains NAL, Nalidixic acid; CIP, ciprofloxacin.

**Strain**	**NAL MIC (mg l^−1^)**	**CIP MIC (mg l^−1^)**	ESBL*	***qnrS* PCR**
*E. coli* TOP10	1.5	0.006	−	−
*E. coli* J53-Azi	4	0.008	−	−
*K. pneumoniae* K1HV	8	0.75	−	+
*E. coli* transformant 1 K1HV	4	0.125	−	+
*E. coli* transformant 2 K1HV	4	0.125	−	+
*K. pneumoniae* 18An	>256	>32	+	+
*E. coli* transformant 1 E18An	4	0.125	−	+
*E. coli* transformant 2 E18An	4	0.094	−	+
*E. coli* E66An	>256	>32	+	+
*E. coli* transformant E66An	4	0.125	+	+
*E. coli* transconjugant E66An	16	0.75	+	+

*ESBL production was identified by resistance to ceftazidime (2 mg l^−1^) and confirmed by a double-disc method.

## Abbreviations

- ESBL, extended-spectrum *β*-lactamase
- PMQR, plasmid-mediated quinolone resistance
- RAPD, random amplified polymorphic DNA
